# Molecular Targeting Therapy against EGFR Family in Breast Cancer: Progress and Future Potentials

**DOI:** 10.3390/cancers11121826

**Published:** 2019-11-20

**Authors:** Amaia Eleonora Maennling, Mehmet Kemal Tur, Marcus Niebert, Torsten Klockenbring, Felix Zeppernick, Stefan Gattenlöhner, Ivo Meinhold-Heerlein, Ahmad Fawzi Hussain

**Affiliations:** 1 Department of Gynecology and Obstetrics, University Hospital RWTH Aachen, Pauwelsstrasse 30, 52074 Aachen, Germany; 2 Institute of Pathology, University Hospital Giessen, Justus-Liebig-University Giessen, Langhanssstr. 10, 35392 Giessen, Germany; 3 Department of Pharmacology and Personalised Medicine, Faculty of Health, Medicine and Life Science, Maastricht University, Universiteitssingel 40, 6229 MD Maastricht, The Netherlands; 4 Department of Molecular Cytology and Functional Genomics, Institute of Pathology, University Hospital Giessen, Justus-Liebig-University Giessen, Langhanssstr. 10, 35392 Giessen, Germany; 5 Department of Biological Sensing and Detection, Fraunhofer Institute for Molecular Biology and Applied Ecology IME, Forckenbeckstrasse 6, 52074 Aachen, Germany; 6 Department of Gynecology and Obstetrics, Medical Faculty, Justus-Liebig-University Giessen, Klinikstr. 33, 35392 Giessen, Germany

**Keywords:** epidermal growth factor receptor, antibody, antibody drug conjugate, tyrosine kinase inhibitor, chimeric antigen receptors t cells

## Abstract

The epidermal growth factor receptor (EGFR) family contains four transmembrane tyrosine kinases (EGFR1/ErbB1, Her2/ErbB2, Her3/ErbB3 and Her4/ErbB4) and 13 secreted polypeptide ligands. EGFRs are overexpressed in many solid tumors, including breast, pancreas, head-and-neck, prostate, ovarian, renal, colon, and non-small-cell lung cancer. Such overexpression produces strong stimulation of downstream signaling pathways, which induce cell growth, cell differentiation, cell cycle progression, angiogenesis, cell motility and blocking of apoptosis.The high expression and/or functional activation of EGFRs correlates with the pathogenesis and progression of several cancers, which make them attractive targets for both diagnosis and therapy. Several approaches have been developed to target these receptors and/or the EGFR modulated effects in cancer cells. Most approaches include the development of anti-EGFRs antibodies and/or small-molecule EGFR inhibitors. This review presents the state-of-the-art and future prospects of targeting EGFRs to treat breast cancer.

## 1. Introduction

Despite the significant improvements in breast cancer detection and treatment, it remains the most life-threatening disease in women, with nearly 2.1 million new cases diagnosed in 2018, representing 25% of all cancers in women, and it is still responsible for more than 600,000 deaths annually worldwide [1].

Breast cancer is a heterogeneous disease in which each tumor presents a different receptor expression profile. Most commonly, breast cancer is associated with the dysrelugation of hormone receptors for estrogen (ER) and progesterone (PR) [2]. However, surface tyrosine kinase receptors belonging to the EGFR/ErbB family (which is named for a homologous erythroblastic leukemia viral oncogene) are very often found upregulated in breast cancers [3]. This family comprises ErbB1/EGFR1, ErbB2/Her2, ErbB3/Her3 and ErbB4/Her4. The amount of EGFRs overexpression determines the extent of dysregulation of their natural functions (regulation of cellular proliferation, differentiation, migration and survival) and their use as indicators for clinical outcome have been reported in several tumor types, including breast cancer [4].

Although the prognosis of Her2^+^ breast cancers is poor, one breast cancer type, namely the triple negative breast cancer (TNBC), has an even worse clinical outcome, with a shorter overall survival than other breast cancer subtypes. TNBC is characterized by a reduced expression of estrogen (ER), progesterone (PR) and Her2 receptors [5]. TNBC accounts for up to 20% of breast cancer cases and despite its heterogeneity, three receptors are commonly overexpressed, namely the EGFR1 (in up to 70% of TNBC cases), the epithelial cell adhesion molecule (EpCAM) and chondroitin sulfate proteoglycan 4 (CSPG4) [6,7]. In 25% of the TNBC cases, the EGFR1 over-expression is caused by its gene amplification and not due to activating mutations [8,9].

Currently, there is no targeted therapy approved for TNBC due to the absence of the typically targeted receptors. Therefore, the treatment relies on a combination of surgery, chemotherapy and/or radiation therapy; the most effective therapy is based on chemotherapies using taxane, cisplatin and anthracycline [10,11].

**Figure 1:**
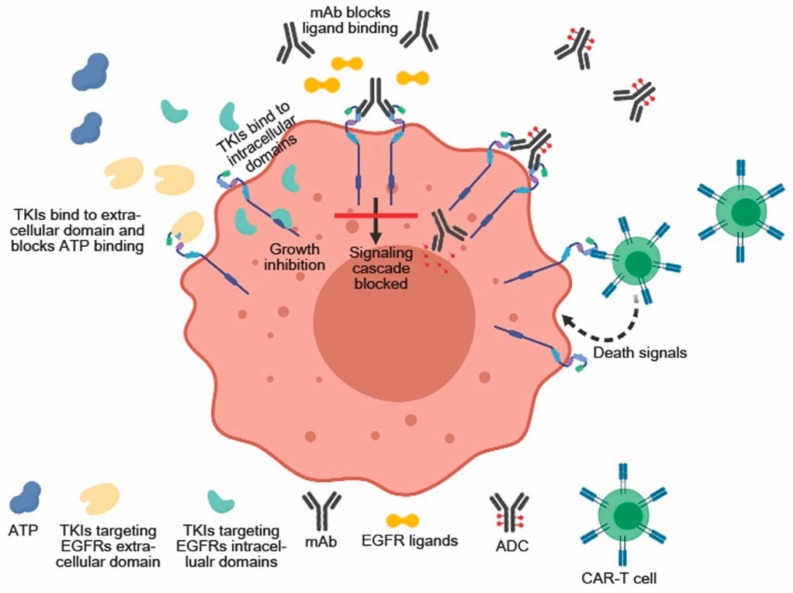
EGFRs targeting therapy. The high expression and/or functional activation of EGFRs have been exploited to generate different therapeutic approaches. These include mAbs, ADCs, tyrosine kinase inhibitors and CAR-T cells.

The correlation between overexpression of EGFR1 and downregulation of Her2 with a poor clinical outcome highlights the need for a targeted therapy for increasing the efficacy and safety profiles of the drugs by aiming for the cancer cells without affecting the healthy cells (Figure 1).

## 2. EGFRs in Breast Cancer

**Figure 2:**
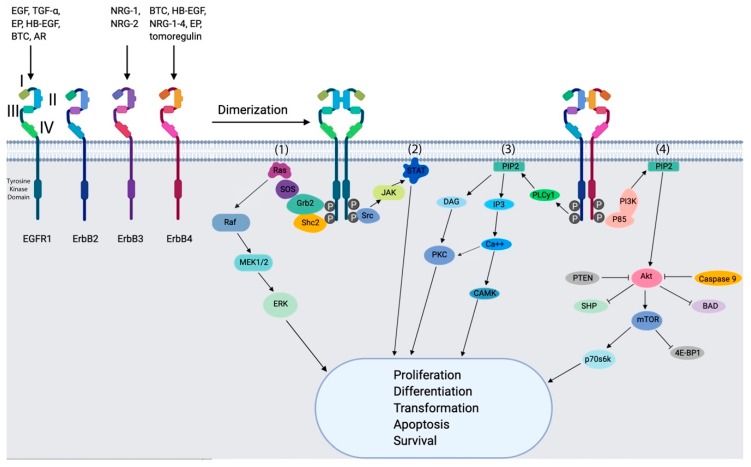
Schematic representation of the four ErbB receptors. The extracellular region of the ErbB receptors EGFR1, ErbB2 (HER2), ErbB3 (HER3) and ErbB4 (HER4) comprises four domains (I–IV). Domains I and III are closely related in sequence, as are domains II and IV. EGFR1 is regulated by peptide ligands, including epidermal growth factor (EGF), transforming growth factor-α (TGF-α), epigen (EP), heparin binding EGF-like growth factor (HB-EGF), betacellulin (BTC) and amphiregulin AR). Activation of the ErbB receptors leads to dimerization, auto- and transphosphorylation of specific tyrosine residues at the C-terminus, recruitment of several intracellular signaling proteins, and subsequent activation of intracellular signaling pathways. (**1**) The RAS-RAF-MEK-ERK pathway: after receptor activation, the complex formed by growth factor receptor-bound protein 2 (Grb2) and son of sevenless (SOS) binds directly or through association of adapter protein Shc2 to specific tyrosine residues on the receptor. This leads to conformational change in SOS, which can recruit and activate Ras. Ras activates Raf, which further activates extracellular regulated kinases (ERK) mediated through mitogen-activated protein kinases (MAPK) MEK1/2. (**2**) The JAK–STAT pathway: the binding of SRC induces when JAKs is recruited through the activation of ErbB receptors. This leads to STAT phosphorylation, which is finally translocated to the nucleus and induce the transcription of specific genes. (**3**) The PLCγ–PKC-CAMK cascade: PLCγ stimulate the production of DAG and IP3 through the activation of PIP2. The binding of DAG and IP3 to the endoplasmic reticulum leads to calcium release and this activate both CaMK and PKC. (**4**) The PIL3-AKT-mTOR signaling cascade: PI3K phosphorylate the phosphatidylinositol-4,5-bisphosphate (PIP2 ) and this leads to the activation of Akt. This signal transfers to intracellular targets such as caspase 9, mammalian target of rapamycin (mTOR), PTEN and SH2 domain-containing inositol 5′-phosphatase (SHP).

Enzymatic phosphorylation of proteins on tyrosine residues is a key element of signal transduction within mammalian cells [12]. In this context, tyrosine kinases exist as integral components of transmembrane receptor molecules and are classified as receptor tyrosine kinases (RTKs). There are several members of this family of RTKs, class I of which includes the EGFRs family. These transmembrane glycoproteins are composed of an extracellular ligand binding domain, a transmembrane domain, and an intracellular receptor domain. They can be activated by 13 known ligands, including EGF, transforming growth factor alpha (TGF-a), amphiregulin (AR), betacellulin (BTC), heparin-binding EGF-like growth factor (HB-EGF), epiregulin (EPR), epigen (EPG) and neuregulins 1–6 (NRG) (Figure 2) [13,14].

Ligand binding induces receptor dimerization, which leads to two modes of signaling. One mode of ErbB signaling is the internalization of the receptor-ligand complex from the plasma membrane, followed by its ubiquitination and degradation of homodimers or recycling of heterodimers to the cell surface. Here, the type of ligand at least partially selects the receptors route to degradation or recycling [13,15].

Following receptor internalization and endocytic sorting, the ErbB receptors translocate into the nucleus either as intact receptors (EGFR1, Her2 and Her3) or as truncated receptors (Her4) due to the presence of nuclear localization signals (NLSs) within the receptors. The ErbB receptors then interact with DNA binding transcription factors such as E2F1, STAT3, and STAT5A or bind to AT-rich sequences of the promoters in order to control the expression of the target genes [16,17,18,19,20]. Another interaction partner of EGFR1 is the RNA helicase A (RHA), that regulates EGFRs target genes in the nucleus of the cancer cells at a transcriptional level.

In the second mode, dimerized receptors provide a scaffold for the subsequent binding and activation of adaptor and effector proteins. The activated proteins dissociate and stimulate signaling cascades, including the KRAS-BRAF-MEK-ERK pathway, phosphoinositide 3-kinase (PI3K), the phospholipase C gamma protein pathway, the anti-apoptotic AKT kinase pathway and the STAT signaling pathway. These pathways induce cell proliferation, angiogenesis and migration and thus facilitate metastasis of the tumors (Figure 2) [14,21]. In addition to the cross-talk between the EGFRs that enable metastasis, cancer also benefits from the interaction of EGFRs with other RTKs, such as IGF-1R and c-MET, that probably account for resistance against EGFRs-targeted therapies [22].

The EGFRs are known to be frequently involved in driving the proliferation and survival of tumor cells [23,24]. One mechanism by which this can occur is over-ovexpression of the receptor at the protein level, for example, as a result of gene amplification. This has been observed in many common human cancers, such as non-small cell lung cancers (NSCLCs), including adenocarcinomas [25] as well as other cancers of the lung [26].

EGFRs are markedly overexpressed across a large variety of epithelial cancers and some immunohistochemical studies have demonstrated that EGFRs expression is associated with poor prognosis [24]. In addition to overexpression, it is recognized that there is potential for deregulated EGFR1 signaling in tumors via a number of alternative mechanisms, including EGFR1 mutations, increased ligand expression and enhanced autocrine loop and heterodimerisation with other EGFRs [27].

### 2.1. EGFR1

All EGFRs are expressed in breast cancer and the ligands of EGFR1, Her3 and Her4 are also frequently detected in this cancer. Her2, on the other hand, does not bind to any of the known ligands [28] and is activated through heterodimerization with other EGFRs. EGFR1 is over-expressed in up to 14% of breast tumors, caused by the amplification of the EGFR gene. An EGFR1 overexpression can also be triggered by missense mutations, with a higher incidence in hereditary breast cancer than in sporadic tumors [29,30].

### 2.2. ErbB2/Her2

Her2 is over-expressed in approximately 20% of all breast cancers through the conversion of the Her2 proto-oncogene into the Her2 oncogene [31,32]. This biomarker stands for more aggressive cancers when compared to Her2-negative breast cancers with features like drug resistance, rapid spreading and higher metastasis [33,34].

### 2.3. ErbB3/Her3

ErbB3/Her3 has an impaired intrinsic kinase activity and requires other ErbB receptors for signaling purposes. After the binding of the high affinity Her3-ligands Neuregulin-1 or Neuregulin-2, Her3 heterodimerizes with EGFR1, Her2 or Her4 or with other RTKs such as fibroblast growth factor receptor (FGFR2) and hepatocyte growth factor receptor (HGFR) for further signaling [35,36]. In estrogen receptor (ER) positive breast cancers, accounting for 80% of all breast cancers, Her3 is mutated in up to 14% and its upregulation appears to be responsible for neuregulin-mediated resistance to Fulvestrant, an ER downregulator [37,38,39]. Her3 is commonly co-expressed with Her2 in breast cancer [40]; it is crucial for luminal mammary epithelium cell viability [41] and therefore, plays a pivotal role in cell growth and survival in Her2-dependent breast cancers [42]. This is mainly because Her2 is incapable of directly activating PI3K signaling, which is then compensated by Her3 phosphorylation and further activation of PI3K [43]. Furthermore, Her3 mutations are not only associated with resistance to ER-targeted therapies but also with resistance to EGFR1- and Her2-directed treatments [44].

### 2.4. ErbB4/Her4

Contrary to the rest of EGFRs, an increase in survival rate and a reduction of proliferation indices were observed with Her4 homodimer expression [45,46]. The correlation between Her4 expression and survival prognosis depends on the location of Her4: membranous Her4 is associated with a good prognosis, whereas nuclear Her4 is associated with lower survival rates [47]. Although homodimerization of Her4 appears to be protective by inducing growth arrest and differentiation, its heterodimerization with other EGFRs promotes cell proliferation [48,49,50].

As EGFRs are involved in cellular transformation, a number of small molecule inhibitors are developed, particularly inhibitors of EGFR1 and Her2, demonstrating anti-proliferative action using small molecule inhibitors or inhibitory antibodies. It has been demonstrated that when antibodies against EGFRs blocking EGF and TGF-a binding to the receptor, this causes inhibition of tumor cell proliferation. In view of these findings, a number of monoclonal antibodies have been developed [51].

Since Her2 expression levels in normal and tumor cells are significantly different and thus, an ideal target for tumor immunotherapy, Her2-targeted anticancer drugs were approved by the FDA in 1998, the first for an anti-Her2 humanized monoclonal antibody (Trastuzumab). Now, the use of Trastuzumab in combination with chemotherapy drugs has become an internationally recognized treatment for advanced Her2^+^ metastatic breast cancer [52].

## 3. Anti-EGFRs Monoclonal Antibodies (mAbs)

mAbs that bind specifically to the extracellular domain of EGFRs were designed to attack cancer on two different routes. mAbs binding the extracellular domain ideally inhibit ligand binding and thus block receptor dimerization, auto-phosphorylation and downstream signaling as well as inducing receptor internalization, degradation and stable downregulation. Alternatively, mABs have been coupled to cytotoxic agents. Thus, if they are co-internalized with the receptor, they induce cell death and thus, generate cancer treatments with high selectivity, toxicity and improved therapeutic windows (Table 1) [53].

### 3.1. mAbs against EGFR1

#### 3.1.1. Cetuximab (Erbitux™)

Cetuximab (Erbitux™) is a chimeric mAb approved in 2004 by the FDA for treating EGFR^+^ metastatic colorectal carcinoma in patients who do not tolerate irinotecan-based therapy [54]. It inhibits ligand mediated receptor tyrosine kinase activation by binding with a high affinity to EGFR1. Cetuximab also mediates receptor down-regulation through the antibody-induced dimerization, resulting in growth inhibition. A phase I trial revealed that combining cetuximab with paclitaxel in advanced breast cancer led to an increased efficacy compared to paclitaxel on its own. Because a high skin toxicity accompanied the greater efficacy observed, this combination was not further considered for treating breast cancer [55].

#### 3.1.2. Panitumumab (Vectibix™)

Panitumumab (Vectibix ™), as a completly human-derived monoclonal antibody, does not contain any xenogenic protein sequences that may trigger an unwanted immune response against the mAb. Binding of Panitumumab to EGFR1 inhibits the signaling cascade and blocks proliferation. It was approved by the FDA in 2006 for the treatment of EGFR-positive metastatic colorectal carcinoma resistant to oxalaplatin-, irinotecan- and fluoropyrimidine based therapies [56]. In a phase II clinical trial, 80% of overall response for TNBC patients was achieved when panitumubab was combined with 5-fluorouracil, epidoxorubicin/cyclophosphamide and docetaxel as neoadjuvant therapy [57]. Pilot studies on the combination of panitumumab and cetuximab with taxane-anthracycline-containing regimens when treating operable TNBC showed a greater efficiency when compared to each treatment on its own [58,59].

#### 3.1.3. Zalutumumab

Zalutumumab is a human mAb with a high affinity to EGFR1 and is characterized by its low immunogenicity which improves its tolerance while retaining the induction of the antibody-dependent cellular toxicity (ADCC) [60,61]. Clinical trials investigating this antibody for the treatment of non-small cell lung cancer (NSCLC) and colorectal cancer were performed. A first phase I+II trial in patients with recurrent disease reported that this antibody was well tolerated, although a skin rash was reported in over 50% of the study participants [60]. A synergistic effect of combining monoclonal antibodies with chemotherapy has been demonstrated by different working groups. Baselga et al. (2013) measured an increased overall response in patients with TNBC when treated with cisplatin and cetuximab compared to those treated with only cisplatin [62]. Carey et al. (2012) showed that combining carboplatin with cetuximab lead to a greater efficacy compared to the efficiency of carboplatin on its own [63].

### 3.2. mAbs against Her2

#### 3.2.1. Trastuzumab (Herceptin™)

Trastuzumab (Herceptin™) is a first generation recombinant humanized antibody that was approved in 1998 for Her2-targeted therapy [64,65,66]. It effectively downregulates the PI3K/Akt and MAPK pathways, leading to improved response and survival rate of Her2-positive breast cancer patients. Furthermore, combining Trastuzumab with different chemotherapeutic agents, such as docetaxel or vinorelbine, reduced recurrence and death in breast cancer patients [66,67,68,69].

#### 3.2.2. Pertuzumab (Omnitarg™)

Pertuzumab (Omnitarg™) is a second generation recombinant humanized antibody that was approved for clinical use in 2012 and significantly promotes survival rates in Her2-positive breast cancer patients. It binds to the binding pocket required for receptor heterodimerisation, thereby inhibiting ligand-induced heterodimerization of corresponding signaling pathways. A synergistic effect of pertuzumab and trastuzumab in Her2-overexpressing cancer cells was observed so that pertuzumab was approved as a neoadjuvant treatment in combination to trastuzumab [70,71,72,73]. However, different studies have reported primary and acquired resistance to trastuzumab and pertuzumab [64,65,74].

#### 3.2.3. Trastuzumab-emtansine (T-DM1, Kadcyla™)

Trastuzumab-emtansine (T-DM1, Kadcyla™) is an antibody-drug conjugate (ADC) composed of the antibody Trastuzumab and the cytotoxic agent DM1 with anti-microtubule activity. T-DM1 was developed to overcome drug resistance in Her2^+^ breast cancer and approved in 2013 as a Her2-targeted therapy for metastatic breast cancer. Although it shows a significantly increased activity compared to trastuzumab on its own, resistance development against it occurs after long-term treatment. Wang and colleagues (2018) showed that the resistance to T-DM1 is induced by STAT3 activation through LIFR overexpression, a receptor tyrosine kinase involved in growth promotion, cell differentiation and other biological processes [75]. Aberrantly activated STAT3 leads to apoptosis resistance by upregulating Bcl-2, Bcl-xL and Mcl-1, among other proteins with anti-apoptotic effects [76]. In Her2^+^ breast cancer, STAT3 also promotes the epithelial-mesenchymal transition (EMT) and resistance to anti-microtubule agents [77]. These findings suggest the need for an STAT3-inhibitor when implementing T-DM1 to treat Her2^+^ breast cancer for preventing resistance after long-term T-DM1 therapy [75].

#### 3.2.4. Trastuzumab Deruxtecan (DS-8201a)

Trastuzumab deruxtecan (DS-8201a) is a antibody-drug conjugate targeting Her2^+^ breast cancer cells. It is a humanized anti-Her2 antibody (Trastuzumab) conjugated with the topoisomerase I inhibitor (exatecan derivative, DXd) through a cathepsins B and L cleavable linker at a drug-to-antibody ratio of 7–8. A phase 1 trail showed that DS-8201a has a high antitumor activity and could significantly reduce tumor growth in intensively pretreated patients. However, this treatment could be associated with some gastrointestinal and hematologic side effects [78].

#### 3.2.5. MEDI4276

MEDI4276 is a bispecific ADC generated to target two different Her2 epitopes by fusing the single chain variable fragments of trastozumab (binds to domain IV of Her2) to the heavy chains of human mAb 39S (binds to domain II of Her2) and conjugating it with tubulysin-based microtubule inhibitor AZ13599185a site-specifically using a cleavable (maleimidocaproyl) linke. During cell mitosis, MEDI4276 is able to inhibit microtubule elongation and induces cell death [79].

The therapeutic and safety properties of MEDI4276 were investigated in a phase I/II clinical trail in pretreated Her2^+^ breast or gastric cancer patients. In this study, the clinical activity of MEDI4276 was confirmed, however, clinical testing has been discontinued due to associated serious adverse events that were reported in 28% of treated patients [79].

### 3.3. mAbs against Her3

As previously mentioned, the correlation between Her3 activation and the resistance to ER-, EGFR1 or Her2-directed therapies highlights the importance of simultaneously targeting the overexpressed receptor (e.g., EGFR1) that causes cancer development, and Her3, which is activated when the patients become resistant against the first treatment by increasing neuregulin (NRG) levels that result in Her3 expression.

#### 3.3.1. Seribantumab (MM-121)

Seribantumab (MM-121) is a humanized mAb that prevents the binding of NRG1 with Her3, leading to the internalization and degradation of the target receptor. This mAb is currently under investigation in different clinical trials for diverse types of cancer, including different subtypes of breast cancer [80]. A phase II trial that addressed the effect of combining seribantumab with exemestane in metastatic heregulin (HR) positive (ER^+^ or PR^+^) breast cancer was stopped due to the significant side effects [81], while a phase II clinical trial examining the effect of combining seribantumab with fulvestrant in NRG-positive, Her2^−^ breast cancer patients is still ongoing [82].

#### 3.3.2. Lumretuzumab (RG7116)

Lumretuzumab (RG7116) is a humanized anti-Her3 antibody that, similarly to seribantumab, prevents the binding of NRG and induces antibody-dependent cell-mediated cytotoxicity [83]. The effect of lumretuzumab, when combined with pertuzumab and paclitaxel on metastatic breast tumors expressing simultaneously Her2- and Her3, has been investigated in a clinical trial phase I. The results of this study revealed that combination therapy has a narrow therapeutic window to promote further clinical tests [84].

#### 3.3.3. MM-111

MM-111 is a bispecific antibody that simultaneously binds to Her3 and Her2 and results in an inhibition of the PI3K/pathway [85]. The safety and clinical effect of MM-111 combined with Trastuzumab are now being investigated in Her2^+^ and NRG^+^ breast cancer in a different phase I/II clinical trial [86].

#### 3.3.4. MCL-128

MCL-128 is also a bispecific antibody that targets Her2 and Her3. A phase II clinical trial was initiated in 2017 to determine its clinical efficiency and safety in combination with chemotherapy and trastuzumab in Her2^+^ metastatic breast cancer or when combined with endocrine treatment in ER^+^ breast tumors [87].

### 3.4. mAs against Her4

Due to the under- and overexpression of Her4 under different scenarios in cancer, the biological importance of Her4 expression in cancer development is not completely understood [88] and the development of treatments targeting Her4 is not as advanced as for targeting EGFR or Her2. The discrepancies in Her4 expression might be due to its four different isoforms, two of them differing in the cytoplasmic domain and the other two in the extracellular region [89].

## 4. EGFRs Small Molecule Inhibitors (RTK Inhibitors)

As EGFRs are involved in cellular transformation, a number of small molecule inhibitors binding to the receptor tyrosine kinases have been developed (Table 1). Although mAbs achieve higher success rates in clinical trials when compared to tyrosine kinase inhibitors (TKIs) and also show a higher specificity as well as a lower toxicity, their production costs are higher and they can only inhibit extracellular targets, whereas the tyrosine kinase inhibitors can target both intra- and extracellular domains of EGFRs [86].

TKIs are not only targeting cells that overexpressing a specific receptor, such as when using monoclonal antibodies, but also cells having mutated or truncated receptors that are constitutively expressed [90].

### 4.1. TKIs against EGFR1

#### 4.1.1. Gefitinib

Gefitinib is a reversible EGFR tyrosine kinase inhibitor with a 200-fold greater affinity to EGFR compared to other ErbB receptors. It has been approved for treating ER^+^ breast cancer and NSCLC patients that are not responding to chemotherapies. In vivo with significant anti-proliferative effects for NSCLC, breast and ovarian cancer as well as prostate and colorectal carcinoma [91,92]. Different clinical trials have shown, that the cell proliferation of tamoxifen-resenstant breast cancer cells expressing ER and lacking PR was decreased when patients were treated with both tamoxifen and gefitinib [93].

#### 4.1.2. Erlotinib (Tarceva™)

Erlotinib (Tarceva™) is a reversible EGFR tyrosine kinase inhibitor approved by the FDA for patients with NSCLC and for the treatment of metastatic pancreatic cancer. Like gefitinib, erlotinib also acts as an ATP analogue by competing with the ATP binding pockets within the EGFR receptors. In vitro, it blocks cell-cycle progression as well as cell proliferation and induces the apoptosis of the target cells [94,95]. Combined with docetaxel, erlotinib increased the response to treatment when compared to the cytostatic agent on its own [96]. Corkery and co-workers (2009) have also reported an anti-proliferative effect of combining erlotinib and gefitinib with docetaxel or carboplatin for treating TNBC cell lines [97]. El Guerrab et al. (2016) also investigated the synergistic effect of the currently FDA-approved mABs cetuximab and panutimab and TKIs gefitinib and erlotinib in the EGFR-overexpressing breast cancer cell lines MDA-MB-468 and MDA-MB-231. mABs were only active on MDA-MB-468, whereas both cell lines were sensitive to the TKIs. The observed sensitivity against the tested EGFR inhibitors did not correlate with the expression levels of EGFR. This was rather caused, in part, by the cell cycle arrest at the G1 phase, followed by apoptosis. Cetuximab and panitumumab were not able to inhibit the RAS/MAPK pathway in MDA-MB-231, whereas in MDA-MB-468, EGFR and ERK phosphorylation was inhibited by the mABs. The observed cell cycle arrest was a consequence of the constitutive expression of ERK 1/2 proteins, showing a link between the sensitivity towards EGFR inhibitors and ERK inhibition [98]. This means that the observed response to these main anti-EGFR therapies was a result of the overexpression of genes implicated in RAS/MAPK and PI3K/AKT pathways and the down-regulation of cyclin genes involved in cell cycle producing resistant cells [98].

### 4.2. TKIs against Her2

#### 4.2.1. Lapatinib (Tykerb or Tyverb™)

Lapatinib (Tykerb or Tyverb™) is a dual reversible TKI for EGFR and Her2 that reacts with the ATP binding site as an ATP analogue inhibiting phosphorylation of EGFR and Her2 and thus prevents the activation of their downstream proliferative pathways, leading to the improvement of progression-free survival [70,99]. This inhibitor was approved in March 2007 by the FDA for the therapy of metastatic breast cancer [100]. Similarly to pertuzumab, lapatinib has also been used in combination with trastuzumab to treat Her2-positive breast tumors; an enhanced apoptosis-induction in vitro was observed [99,101,102]. The high levels of endogenous ATP induce drug resistance to reversible TKIs such as lapatinib, gefitinib and erlotinib. This indicates the need for more effective inhibitors driving the development of irreversible inhibitors [103].

#### 4.2.2. Neratinib (Nerlynx™)

Neratinib (Nerlynx™) is an irreversible TKI that as well as lapatinib, inhibits the interaction of the tyrosine kinase with ATP and therefore, the receptor phosphorylation. This TKI also inhibits EGFR and Her4 and was approved in July 2017 by the FDA as an adjuvant to treat early stage Her2-positive breast cancer following trastuzumab therapy [104,105]. Canonici and co-workers (2013) reported the synergistic effect of trastuzumab. Combined with neratinib, Trastuzumab was more efficient in sensitive and acquired-resistant Her2-overexpressing cell lines than on its own when reducing cell viability. Resistance to trastuzumab is then overcome by targeting both the intracellular (neratinib) and extracellular (trastuzumab) domain of Her2 [106]. Neratinib has also shown promising results on its own in Her2-positive breast cancer clinical trials [104,107].

Still, breast cancer patients carrying Her2-overexpression are, in approx. 70% of the cases, innate or acquired-resistant to Her2-targeted therapies [108].

Breslin et al. (2017) developed neratinib-resistant Her2-positive breast cancer cells and confirmed their bi-directional cross-resistance to the Her2-targeting drugs trastuzumab, lapatinib and afatinib. These data suggest that if a Her2-positive tumor was resistant to neritinib, the efficacy of other treatments targeting this receptor would be compromised due to the prior exposure to neritinib. In the same way, drug-naïve patients would respond better to nerotinib than those treated with other Her2 drugs first [109]. Whether neratinib overcomes the resistance to other antibodies targeting Her2 or potentiates it depends on each individual, which highlights the importance of a personalized therapy. Other irreversible TKIs that are active during clinical studies in Her2-positive breast cancer patients are afatinib (BIBW2992) and dacomitinib (PF-00299804) [110].

### 4.3. TKIs against Her3

Due to the inactivity of the kinase in Her3 and thus to the requirement of a heterodimerization with other ErbB receptors for its activation, blocking the receptor partners leads to the suppression of Her3 activity [111]. This means that TKIs that inhibit other EGFRs indirectly act as Her3 inhibitors as well.

### 4.4. TKIs against Her4

#### 4.4.1. Canertinib (CI-1033) 

Canertinib (CI-1033) is a first-generation pan-ErbB inhibitor that was developed by Pfizer to inhibit EGFR, Her2 and Her4. Because several clinical trials in phase I or II reported its limited effect, this drug was not further developed [112].

#### 4.4.2. Afatinib (Gilotrif™)

Afatinib (Gilotrif™) is an irreversible TKI that simultaneously targets EFR, Her2 and Her4 [113]. Due to the inhibition of the interaction partners, afatinib also suppresses the Her3-mediated signaling [111]. De Pauw and colleagues (2018) investigated the synergistic effect of afatinib and cetuximab in cetuximab-sensitive and -resistant head and neck squamous cell carcinoma (HNSCC) cell lines. In this study, Afatinib overcame intrinsic and acquired cetuximab resistance by inducing cell cycle arrest and thus, apoptosis. In preclinical trials, afatinib achieved a sensitization towards radiotherapy in HNSCC and pancreatic cancer cells [114,115].

## 5. Chimeric Antigen Receptor (CAR) Cell-Based EGFRs Targeting Therapy

**Figure 3:**
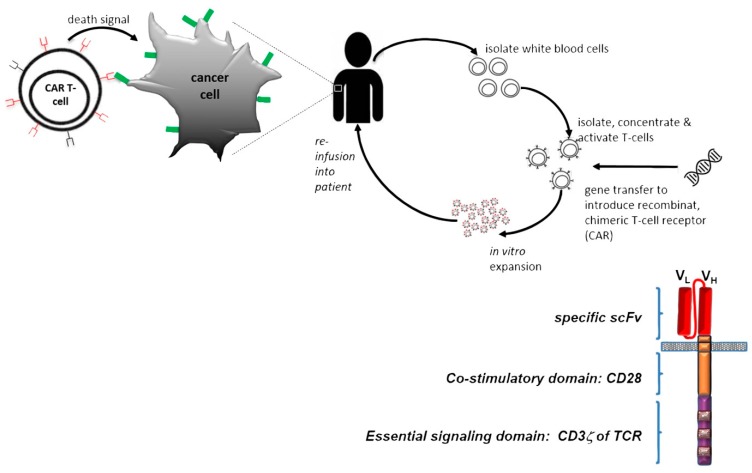
Schematic representation of the mechanism and structure of CARs. For adoptive cell immunotherapy, patients’ own T cells are collected to be modified with specific CAR using a viral vector system. After in vitro expansion, the CAR-T cells are infused back to the patients to bind and lyse the targeted tumor cells. The recombinant T cells consist of an extracellular targeting region, a transmembrane region and various intracellular signalling domains. The extracellular section is a single-chain variable fragment (scFv) and the intracellular section is the activation domains which include CD3 zeta chain and the CD28 co-stimulatory domain, which can enhance the signal transduction and affect their functions.

Chimeric antigen receptors (CARs) are synthetic constructs that are designed to be expressed in host T cells or NK cells and to induce an immune response against a specific target antigen and cells expressing that antigen independently of presentation by the Major Histocompatibility Complex (MHC) [116]. The CAR typically comprises an antibody fragment, such as a scFv or Fab fragment of a monoclonal antibody, incorporated in a fusion protein that also comprises additional components, such as a CD3-zeta or CD28 transmembrane domain and selective cell activating moieties, including the endodomains of CD3-C, CD28, OX40, 4-1BB and DAP10 (Figure 3).

A variety of CAR-T or CAR-NK cell-based adoptive immunotherapy has been developed for hematopoietic cancers or solid tumors therapy [117,118]. Successful application of T cell immunotherapy for late-stage breast cancer has been published very recently [119]. Engineered T cells showed complete durable remission of malignant disease after being treated with autologous tumor-infiltrating lymphocytes (TILs) that had been enriched ex vivo for reactivity to neoantigens. Furthermore, a CAR construct-specific for both Her2 and Muc1 had promising in vitro results in a breast cancer model [120] and a dual-target CAR-T cells targeting Her2 and IL13Ra2 showed greater success than single-target CAR-T cells in a xenograft glioma model [121].

Recently, a split, universal, and programmable CAR-T cells pre-targeting system was generated by exploiting the unique folding and specific interaction properties of engineering coiled coils peptide domains (Zippers). Here, a zipper domain was fused to a Her2-specific single antibody chain (scFv) and its specific binding zipper was expressed on the T-cell surface. This system allowed the accumulation of the scFv before the injection of the therapeutic T-cells and therefore, reduced the adverse effects which could associate with CAR-T cell therapies [122].

## 6. Anti-EGFR Agent-Associated Toxicity (Side Effects of EGFR Inhibitors)

Although many monoclonal antibodies have been approved for treating different cancer types, they also carry drawbacks as therapeutic and diagnostic (theranostic) tools. For diagnostic purposes, their size provokes a slow blood clearance, resulting in an increased exposure of patients to the radionuclides and therefore, an increase of side effects and preventing an optimal visualization of the tumors [123]. The size of the antibodies also leads to slow extravasation from the blood and poor tissue penetration and thus, to higher antibody concentrations needed for therapeutic purposes, which can result in non-desired immunological responses [124]. Some of the side effects caused by the monoclonal antibodies are severe acne, hypotension, cardiac arrest and toxicity, dizziness, bronchospasm, among others [90]. Nevertheless, lower concentrations of mABs are required to achieve inhibition when compared to TKIs and are less toxic than the synthetic therapeutics.

TKIs have lower production costs compared to mABs, but their efficiency in clinical trials is lower compared to the biological compounds. Side effects of TKI-based therapies are, e.g., skin rash, bone pain, stomatitis and pneumonitis [90].

Although both approaches have their advantages when compared with each other, with mAB displaying a higher specificity and irreversible TKI a more sustained action, these formats have to be improved to improve their efficiency and reduce their side effects for cancer treatment.

## 7. Novel Strategies

Photoimmunotherapy (PIT) or near-infrared (NIR) PIT is an oncological treatment that combines photodynamic therapy of tumors with immunotherapy treatment. Combining photodynamic therapy with immunotherapy enhances the immunostimulating response and has synergistic effects for metastatic cancer treatment [125,126].

### 7.1. Cetuximab-IR700 

Cetuximab-IR700 is a antibody-photosensitizer conjugate (APC) of EGFR1 targeting mAb Cetuximab (see 2.1.1 for details) and silicon-phthalocyanine derivative of near-infrared dye IRdye700DX. Cetuximab-IR700 has successfully completed a phase I/II clinical trial for the treatment of inoperable recurrent head and neck cancer an was fast-tracked by the FDA for a phase III trial that is currently underway in the US, Asia and Europe [127].

The same APC is also used for the treatment of TNBC [128]. This study from the Kobayashi lab highlights the importance to optimize the dosing of the APC and the amount of NIR light to achieve maximal efficacy with minimal side effects [128].

### 7.2. Cytotoxicity and Immunotherapy

Cytotoxicity and immunotherapy of NIR-PIT is not accomplished by ROS production. Instead, photochemical reactions within the dye IRdye700DX lead to physiochemical reactions in the vicinity of the mAb-dye-conjugate that ultimately lead to the breakdown of cell membrane integrity, water influx and subsequent cell death. This cell death recruits immature dendritic cells within the tumor microenvironment. Days after the treatment, analyses revealed an increased number of CD8^+^ T-cells reacting to different tumor-specific antigens than before the treatment. A more detailed discussion of NIR-PIT is available in [129].

## 8. Conclusions and Perspectives

In recent decades, the pivotal role of EGFRs for the activation of downstream signaling pathways during cancer development has been elucidated. Many monoclonal antibodies and tyrosine kinase inhibitors targeting EGFRs are currently under investigation, in preclinical development, or have been approved for treating cancer. Although clinical studies reveal promising results when using both approaches, a many patients are resistant to these treatments by activating or overexpressing alternative pathways and present a more aggressive phenotype, with more migratory and invasive cells compared to the non-resistant tumors. In addition, general understanding of tumor clonality and the implications of tumor evolution during cancer treatment may help to optimize the outcome of cancer patients.

The documented innate or acquired-resistances against EGFR-targeted therapies as well as the reported cross-resistances within the treatments highlight the urgency of understanding the interplay between the ErbB receptors and their activated pathways to modulate them as diagnostic and therapeutic tools during cancer.

Photoimmunotherapy (antibody photosensitizer conjugate) represents an efficient strategy to overcome the resistance and side effects of the developed mAbs and TKIs, creating treatments with higher efficiency and safety profiles.

## Figures and Tables

**Table 1:** Targeting therapy against EGFR family in breast cancer under investigation for treatment of breast cancer.

Drug	Format	Target	Clinical Phase	Literature
Cetuximab	Chimeric MAb	EGFR1	I, II	[44,55]
Panitumumab	Humanized MAb	EGFR1	II	[56,57]
Zalutumumab	Human MAb	EGFR1	I, II	[60,61]
Trastuzumab	Humanized MAb	HER2	approved	[64,65,66]
Pertuzumab	Humanized MAb	HER2	approved	[70,71,72,73]
Trastuzumab Emtansin	Antibody-drug conjugate	HER2	approved	[75,76]
Trastuzumab Deruxtecan	Antibody-drug conjugate	HER2/Tubulin	I, II	[78]
MEDI4276	Bispecific antibody	HER2	I, II	[79]
Seribantumab	Humanized MAb	HER3	I, II	[80,81,82]
Lumretuzumab	Humanized MAb	HER3	I	[83,84]
MM-111	Bispecific antibody	HER3	I, II	[85,86]
MCL-128	Bispecific antibody	HER2/3	I, II	[87]
Gefitinib	Small-molecule tyrosine kinase inhibitor	EGFR1	approved	[91,92]
Erlotinib	Small-molecule tyrosine kinase inhibitor	EGFR1	approved	[94,95,98,99]
Lapatinib	Small-molecule tyrosine kinase inhibitor	EGFR1/HER2	approved	[70,100,101]
Neratinib	Small-molecule tyrosine kinase inhibitor	HER2	approved	[105,106,108]
Canertinib	Small-molecule tyrosine kinase inhibitor	pan-ERB	I, II	[113]
Afatinib	Small-molecule tyrosine kinase inhibitor	EGFR1, HER2, HER4	approved	[114,115,116]
